# Fluorescent Labeling Strategies for Tracking Micro- and Nanoplastics in Biological Systems

**DOI:** 10.3390/ma19183943

**Published:** 2026-09-17

**Authors:** Charles E. Bardawil, Jarrett Dobbins, Alexander C. Dodson, Shannon Lankford, Cedric Schaack, Rajeev Dhupar

**Affiliations:** 1 Department of Cardiothoracic Surgery, Wake Forest University School of Medicine, Winston-Salem, NC 27157, USAdavid.j.dobbins@wfusm.edu (J.D.); alexander.dodson@wfusm.edu (A.C.D.); shannon.lankford@advocatehealth.org (S.L.); 2 Department of Chemistry, Wake Forest University, Winston-Salem, NC 27109, USA; 3 Center for Functional Materials, Wake Forest University, Winston-Salem, NC 27109, USA; 4 Surgical and Research Services Division, VA Pittsburgh Healthcare System, Pittsburgh, PA 15212, USA

**Keywords:** microplastics, nanoplastics, fluorescence, microscopy, spectroscopy

## Abstract

Micro- and nanoplastic (MNP) contamination is a global health issue, with growing concerns regarding human exposure and potential health impacts. Understanding their biodistribution, cellular uptake, and toxicological effects in biological systems requires sensitive analytical tools capable of detecting and localizing particles across multiple scales and matrix compositions. Techniques such as Fourier-transform infrared spectroscopy, Raman spectroscopy, and mass spectrometry provide valuable chemical information but face limitations in nanoscale detection, sensitivity, tissue penetration, and spatial localization within biological matrices. Fluorescent labeling techniques have emerged as a powerful complementary approach, offering high sensitivity, real-time imaging capability, and broad compatibility with in vitro and in vivo platforms. This review summarizes the principal fluorescent labeling strategies used for MNPs, including adsorption-based staining, swelling-diffusion methods, covalent conjugation, and polymerization-based incorporation of fluorescent probes. We also examine the imaging modalities used to visualize and quantify fluorescent MNPs in biological contexts, including fluorescence microscopy, confocal microscopy, flow cytometry, and whole-body optical imaging. Applications in cellular uptake studies, biodistribution in animal models, transport across biological barriers, and cumulative accumulation measurements are highlighted. Persistent challenges such as dye leaching, biological autofluorescence, photobleaching, and polymer-dependent labeling efficiency are addressed, alongside emerging opportunities in near-infrared fluorescence imaging and multimodal detection strategies. Continued development of fluorescent labeling approaches will enhance our ability to track MNPs across biological systems and inform our understanding of their toxicological consequences.

## 1. Introduction

Plastics are synthetic polymeric materials produced from petroleum- or natural gas-derived monomers and commonly formulated with additives that modify their mechanical, thermal, and chemical properties [1]. Environmental weathering, mechanical abrasion, and degradation progressively fragment larger plastic materials into micro- (1 µm–5 mm) and nanoplastics (1 nm–1 µm; MNPs). These particles are now pervasive across environmental compartments and can enter food webs and respiratory environments relevant to human exposure [2]. Growing concern regarding the potential biological effects of MNP exposure has increased the need for analytical methods that can identify, localize, and quantify particles in laboratory models and biological specimens [3].

The detection of MNPs in biological systems remains technically challenging. Biological matrices contain cells, extracellular matrix components, lipids, proteins, and endogenous fluorescent molecules that can obscure or mimic particle-associated signals. Established analytical methods, including Fourier-transform infrared (FTIR) spectroscopy, Raman spectroscopy, and mass spectrometry, provide important information regarding polymer identity and chemical composition. However, their application to biological samples may be constrained by limited spatial resolution, interference from complex matrices, background fluorescence, sample-processing requirements, or limited ability to provide real-time spatial information within intact cells and tissues [4,5,6,7,8,9,10,11].

Fluorescent labeling offers a complementary strategy for investigating MNP behavior in vivo and in vitro. When a fluorophore remains stably associated with the particle, fluorescence-based approaches can enable rapid visualization, spatial localization, longitudinal tracking, and semi-quantitative comparison of MNP-associated signal across cells, tissues, and whole organisms. Fluorescence can also be integrated with microscopy, flow cytometry, and whole-animal optical imaging platforms that are widely available in biomedical research settings [11]. In addition, fluorescence-guided workflows can identify candidate particles or regions of interest for targeted Raman analysis, thereby improving analytical efficiency and supporting orthogonal confirmation of polymer identity [12].

Importantly, interpretation of fluorescence depends on dye retention, labeling-induced particle changes, the biological matrix, and the detection platform. Noncovalently associated dyes may dissociate from particle surfaces and bind to lipid-rich or other biological structures, whereas covalent, matrix-embedded, and polymerization-based labeling approaches may improve signal stability but can alter particle surface chemistry or environmental representativeness. Therefore, fluorescent labeling must be regarded as an experimental design variable rather than a purely technical detection step.

**Figure 1:**
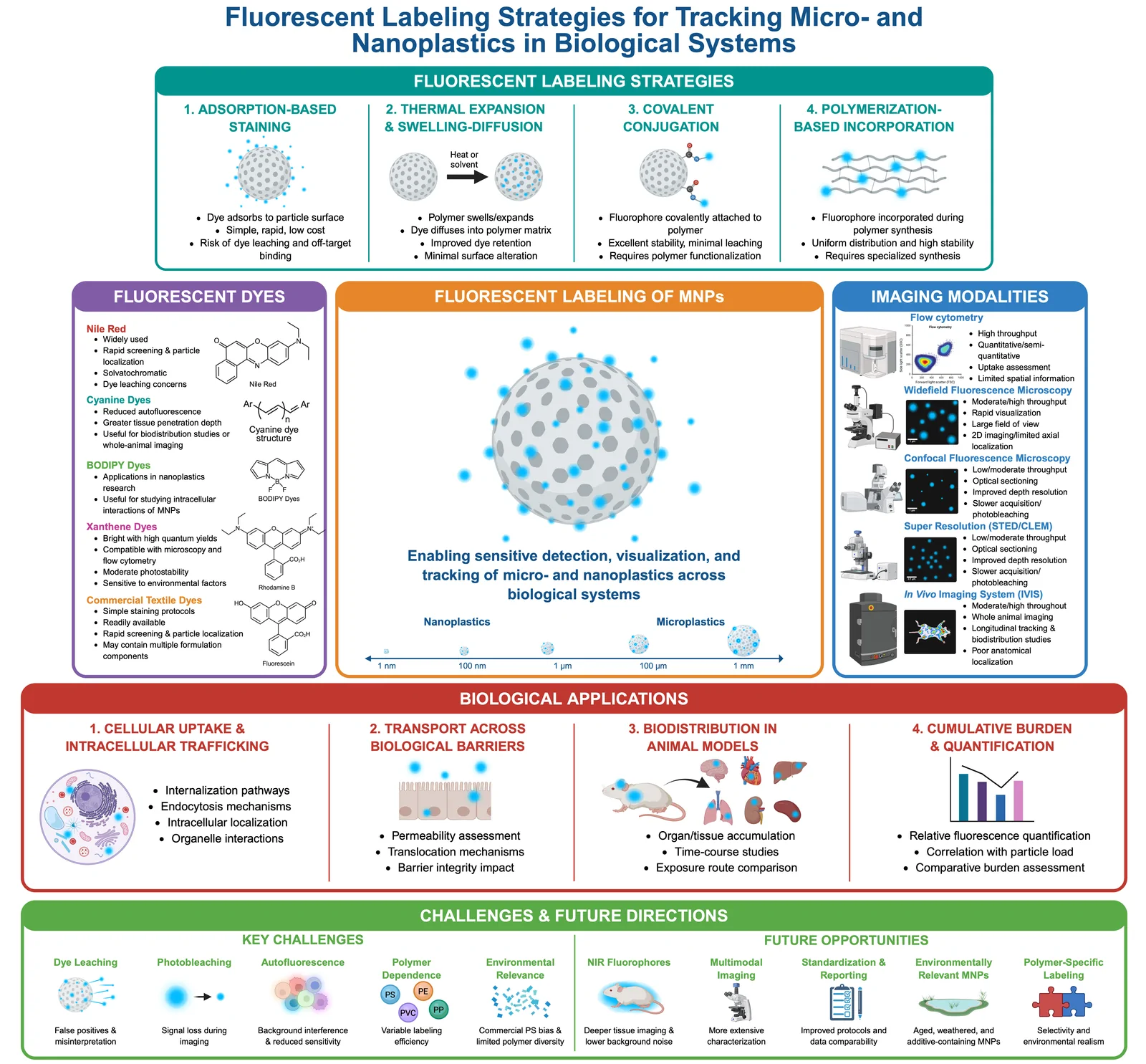
Overview of fluorescent labeling strategies for tracking micro- and nanoplastics in biological systems.

This review examines the principles, strategies, and applications of fluorescent labeling for tracking MNPs in biological systems. We summarize fluorescence fundamentals and major classes of fluorophores used for MNP labeling. We then compare adsorption-based staining, thermal- and solvent-mediated swelling-diffusion, covalent fluorophore conjugation, and polymerization-based incorporation strategies. Subsequent sections review imaging modalities used to detect fluorescent MNPs, including fluorescence and confocal microscopy, flow cytometry, and near-infrared and whole-animal optical imaging. Finally, we discuss biological applications in cellular uptake, intracellular localization, barrier transport, biodistribution, and relative burden assessment, with particular emphasis on the validation requirements, experimental limitations, and emerging approaches needed to improve the biological relevance of fluorescent MNP studies (Figure 1).

## 2. Fluorescent Labeling of MNPs

### 2.1. Fluorescence Considerations

Fluorescence is a photoluminescent process in which a fluorophore absorbs photons at a specific excitation wavelength and subsequently emits light at a longer, lower-energy wavelength as it relaxes from an excited electronic state [13]. The wavelength difference between excitation and emission light is referred to as the Stokes shift. The magnitude of the Stokes shift is important for imaging because it determines the degree of spectral separation between excitation and emission signals. Greater spectral separation can reduce background interference and improve the signal-to-noise ratio, thereby enhancing detection sensitivity [14,15].

Several parameters govern fluorophore performance. Quantum yield describes the efficiency of photon emission relative to photon absorption and directly influences signal intensity [16]. Photostability determines the resistance of fluorophores to photobleaching during repeated or prolonged excitation, which is particularly important for time-course imaging, repeated acquisition, and in vivo applications. Additionally, fluorescence properties are dependent on solvent polarity, pH, and the presence of other molecules that result in quenching [17]. For research in biological systems, these considerations are especially relevant because fluorophores may be embedded within, adsorbed onto, or conjugated to hydrophobic polymer matrices. Such interactions can alter fluorescence emission spectra, signal intensity, photostability, and dye retention.

Tissue optical properties are another consideration when selecting fluorophores. Light scattering, tissue absorption, and autofluorescence from endogenous biomolecules can significantly attenuate or obscure fluorescence signals. These limitations are pronounced in the visible spectrum and have driven increasing interest in near-infrared fluorophores, which offer improved tissue penetration and reduced background signal [18].

### 2.2. Fluorophore Classes

Fluorescent dyes used in MNP research are not interchangeable, and their suitability depends on the research objective, polymer composition, labeling strategy, sample matrix, and detection platform. In addition to relevant photophysical properties, dye polarity, charge, lipophilicity, and molecular size also influence adsorption to or incorporation within different polymers. Consequently, the same fluorophore may exhibit different fluorescence intensity, emission spectra, retention, and photostability depending on the polymer, staining solvent, dye loading, particle size, weathering state, and surrounding medium. Performance demonstrated for one dye-polymer combination should not necessarily be assumed to apply across other MNP types or experimental conditions [19,20,21,22,23,24].

The requirements for dye selection also differ between environmental screening and biological tracking. Environmental screening requires strong contrast between plastics and natural materials, reproducible staining across the polymers of interest, and limited nonspecific fluorescence from organic matter. Because fluorescence color or intensity alone generally cannot establish polymer identity, suspected particles may require confirmation by Raman or FTIR spectroscopy [20,22,23,25]. Biological tracking imposes additional requirements in that the fluorophore must remain associated with the particle for the full study duration, retain adequate signal in the relevant biological medium, produce minimal free-dye or autofluorescent background, and avoid substantially altering particle surface properties or biological behavior [24,26,27,28]. Fluorophores suitable for short-term cellular microscopy may be inappropriate for longitudinal tracking or whole-animal biodistribution, where stable labeling and longer-wavelength emission may be necessary.

#### 2.2.1. Nile Red

Nile Red (λ_ex_ ~540 nm, λ_em_ = 580–640 nm) is among the most extensively evaluated stains in MNP research. It has been applied to the detection and enumeration of microplastics collected from water, sediment, biological samples, and other environmental matrices [20,21,22,23]. The dye associates with hydrophobic polymer surfaces, and its fluorescence intensity and emission color depend on the polymer, staining solvent, dye concentration, excitation wavelength, and detection filter [20,21,22]. Because it is strongly solvatochromic, its excitation and emission spectra vary substantially based on the polarity of the surrounding medium rather than exhibiting fixed maxima; typical operational ranges span λ_ex_ ~480–550 nm and λ_ex_ ~530–640 nm. In hydrophobic polymer matrices or lipid environments, Nile Red exhibits blue-shifted fluorescence, whereas in polar aqueous media, its emission shifts to longer wavelengths and undergoes pronounced quenching.

Shim et al. demonstrated Nile Red staining of several common microplastic polymers and evaluated the effects of staining concentration, incubation time, and excitation wavelength [20]. Maes et al. subsequently developed a rapid screening workflow in which Nile Red-stained particles retained on filters were photographed under blue-light excitation and quantified by image analysis [21]. Erni-Cassola et al. extended this approach to environmental microplastics measuring approximately 20 µm–1 mm [22]. Nile Red staining has also been used to select suspected microplastics for confirmatory micro-Raman analysis, thereby reducing the number of particles requiring spectroscopic examination [25].

Nile Red fluorescence is not specific to plastic. Chitin, wood-derived material, oils, and other organic particles can produce fluorescence or be misclassified as plastic unless organic matter is removed and appropriate controls are included [22,23]. Differences in Nile Red emission can assist with preliminary discrimination among polymers, but fluorescence color alone does not provide definitive polymer identification [20,22,25]. Consequently, Nile Red is most appropriate as a rapid screening or particle-localization method supported by procedural blanks, matrix controls, standardized imaging thresholds, and confirmatory Raman or FTIR spectroscopy [22,23,25].

#### 2.2.2. Xanthene Dyes

Rhodamine B, rhodamine 6G, and fluorescein derivatives have been evaluated directly for fluorescent labeling of MNPs. Rhodamine B (λ_ex_ ~540 nm, λ_em_ = 565–570 nm) staining has been demonstrated for polyethylene (PE), polypropylene (PP), polystyrene (PS), polyvinyl chloride (PVC), and polyurethane microplastics, with staining performance dependent on the polymer and the solvent used to deliver the dye [29]. The method was developed primarily to improve the visibility and fluorescence-based tracking of transparent or white particles in controlled laboratory experiments [29]. Rhodamine dyes are especially attractive because they are generally bright with excitation and emission profiles compatible with common fluorescence microscopy platforms.

Rhodamine 6G (λ_ex_ ~530 nm, λ_em_ = 550–560 nm) has been used to prepare fluorescent nylon and PP microplastics for microscopy-based biological experiments and has also been evaluated for adsorption to PS microplastics and several nanoplastic polymers [30,31,32]. Its response is polymer-dependent. In one spectroscopic study, adsorption to PS produced negligible fluorescence enhancement, whereas detectable spectral changes were observed for high- and low-density PE and PS nanoplastics in another study [30,31]. Therefore, rhodamine 6G fluorescence should not be assumed to respond uniformly across polymer types.

Fluorescein (λ_ex_ ~490 nm, λ_em_ = 515–520 nm) and fluorescein isothiocyanate have also been investigated in MNP labeling studies. Heat-assisted fluorescein isothiocyanate staining was demonstrated for PE, PS, PVC, and polyethylene terephthalate (PET) microplastics [19], while fluorescein sodium salt produced polymer-dependent spectral responses after adsorption to PP, PVC, PE, and PS nanoplastics [31]. These studies indicate that xanthene dye performance depends on the polymer, labeling conditions, and detection method rather than on dye class alone [19,29,30,31]. However, its emission intensity and quantum yield remain sensitive to environmental pH, ionic strength, and specific matrix components, with its photophysical behavior being strongly dictated by pH, transitioning between non-fluorescent neutral/monoanionic states and the highly fluorescent dianion.

#### 2.2.3. Cyanine and Related Polymethine Dyes

The cyanine dyes with the clearest direct application in MNP research are cyanine 3 (Cy3), cyanine 5.5 (Cy5.5) and IRDye 800CW. Cy3 phosphoramidite (λ_ex_ ~555 nm, λ_em_ = 570 nm) was evaluated as an adsorptive label for PP, low- and high-density PE, PS, PET, and PVC nanoplastics [31]. Polymer-dependent shifts in fluorescence spectra enabled differentiation of PVC, PS, and PET under the experimental conditions, although this method did not distinguish every polymer examined.

Cy5.5 carboxylic acid (λ_ex_ ~680 nm, λ_em_ = 695–710 nm) has been incorporated into fragmented PP, PET, and PE microplastics for near-infrared imaging in mice [33,34,35]. Cy5.5-labeled PP microplastics of approximately 5 and 10–50 µm were used for in vivo and ex vivo imaging following oral administration [33]. A heat- and solvent-assisted swelling-diffusion method was subsequently used to label PET microplastics smaller than 10 µm [34]. More recently, Cy5.5 was incorporated into irregular PE microplastics measuring 1–10 and 10–50 µm using solvent-assisted diffusion [35]. Fluorescence retention was evaluated in simulated salivary, gastric, and intestinal fluids before the particles were used for oral-exposure imaging [35]. These studies demonstrate the utility of Cy5.5 for MNP tracking but also show that labeling and retention conditions must be established separately for each polymer.

Near-infrared (NIR) dyes, such as IRDye 800CW (λ_ex_ ~775 nm, λ_em_ = 790–795 nm) offer reduced tissue autofluorescence and improved compatibility with in vivo and ex vivo optical imaging platforms, making them especially relevant for biodistribution and whole-animal imaging studies. IRDye 800CW has been covalently conjugated through its N-hydroxysuccinimide ester to amino-functionalized PS microplastics [32]. The labeled particles were visualized by confocal microscopy and whole-animal near-infrared imaging, including ex vivo organ imaging after intravenous administration [32]. Covalent conjugation reduced reliance on physical adsorption, but purification, free-dye controls, linkage stability, and characterization of labeling-induced changes in particle properties remain necessary. In addition, the reduced fluorescence absorption by organic tissue when using dyes in the NIR range allows for greater imaging depth and improved signal-to-background in tissue.

#### 2.2.4. BODIPY Dyes

BODIPY dyes have been used in a small number of specialized nanoplastic studies rather than as routine environmental stains. Okkelman et al. incorporated a deep-red dibutoxy-aza-BODIPY dye into PS- and poly(methyl methacrylate) (PMMA)-based nanoplastics measuring less than 200 nm [36]. Differences in the fluorescence lifetime of the dye within different polymer matrices enabled fluorescence-lifetime “barcoding” and tracking of nanoplastic interactions with live intestinal organoids [36].

In another application, an iodine-substituted BODIPY photosensitizer was conjugated to amino-functionalized PS nanoplastics and used for photocatalytic proximity labeling of proteins interacting with the particles inside cells [37]. This approach employed BODIPY as a functional photochemical component of a nanoplastic-protein-interaction assay rather than as a general stain for detecting unknown environmental particles. Current MNP-specific evidence supports BODIPY primarily for engineered model nanoplastics and specialized fluorescence or photochemical measurements [36,37].

#### 2.2.5. Other Visible-Spectrum Dyes

Safranine T (λ_ex_ ~520 nm, λ_em_ = 570–577 nm) has been evaluated as a fluorescent label for PE, PS, PVC, and PET microplastics using a heat-assisted staining procedure [19]. In that protocol, particles were incubated with the dye at elevated temperature and subsequently cooled and washed. The authors attributed the improved retention to diffusion of the dye into the heated polymer matrix followed by physical retention during cooling. Heating produced stronger and more persistent fluorescence than room-temperature surface staining under the conditions tested [19]. Further use of Safranine T in MNP research remains limited, and its performance in biological matrices and for longitudinal particle tracking has not been established.

Coumarin 6 (λ_ex_ ~455 nm, λ_em_ = 500–505 nm) has been evaluated directly as a microplastic stain. Cheng et al. optimized a Coumarin 6 method using an acetone-ethanol staining system and demonstrated fluorescence from ten plastic types [38]. Under the selected conditions, a dye concentration of 1 mg/L and an immersion time of 60 min were used, and the method was applied to microplastics recovered from Tokyo Bay seawater [38]. A subsequent comparative study evaluated Coumarin 6 and Coumarin 153 against Nile Red using pristine and mechanically or thermally damaged microplastics. Staining efficiency depended on the combination of dye, polymer, particle condition, and illumination system [39]. PP was among the most difficult polymers to stain consistently, whereas PET generally produced stronger staining responses [39]. Thus, Coumarin 6 is supported as an alternative microplastic stain, but polymer-specific recovery and detection performance must be established for the matrix and imaging configuration being used [38,39].

#### 2.2.6. Commercial Textile Dyes

Commercial textile-dye formulations have been used to fluorescently label microplastic particles and fibers for laboratory exposure studies. Immersion dyes, or iDyes, have been developed and applied as practical staining reagents for laboratory studies [40,41]. These dyes can provide strong fluorescence across multiple polymer types and are compatible with relatively simple staining workflows. Their use is particularly relevant for studies requiring rapid screening or visualization rather than definitive chemical identification. Karakolis et al. demonstrated that selected iDye Poly and Rit DyeMore formulations could label particles and fibers composed of different polymers, providing an accessible method for producing fluorescent microplastics for organism-exposure experiments [41]. Gao et al. subsequently compared four textile-dye formulations with Nile Red across 17 virgin and weathered polymers and found that staining performance varied with polymer type, particle form, dye formulation, temperature, and color channel [42].

Because these studies evaluated commercial dye formulations, the product names should not be treated as discrete molecular dye classes. Such formulations may contain more than one colorant and additional formulation components. Commercial textile dyes are most appropriately used to prepare visible model microplastics for controlled experiments rather than to provide definitive polymer identification in environmental samples.

### 2.3. Fluorescent Labeling Strategies

An intuitive approach to detecting environmental MNPs in biological models might be to apply fluorescent dyes directly to the cells or tissues in situ. However, this strategy is largely ineffective because fluorophores like Nile Red and rhodamine derivatives readily bind to endogenous lipid-rich structures and hydrophobic proteins, causing substantial background interference. Instead, researchers must utilize pre-labeling strategies that stably bind the dye to MNPs before they ever contact the biological matrix.

Multiple fluorescent labeling strategies exist for MNPs, each with distinct advantages and limitations related to label stability, dye leaching, polymer compatibility, preservation of particle properties, and biological applicability. No single strategy is optimal for all experimental settings. Adsorption-based staining may be appropriate for rapid screening or short-term in vitro studies, whereas longitudinal cellular-trafficking and in vivo biodistribution experiments generally require more stable matrix-embedded, covalent, or polymerization-based labels. 

#### 2.3.1. Adsorption-Based Staining

**Figure 2:**
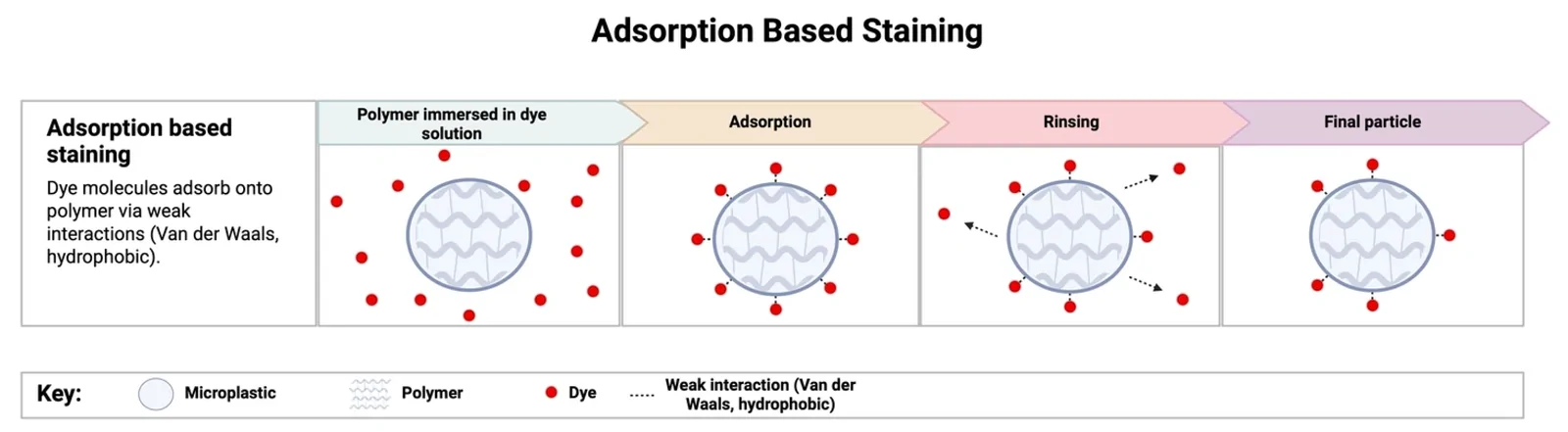
Surface-associated dye provides fluorescent labeling of the polymer.

Adsorption is the accumulation of molecules at an interface which results in a surface concentration that differs from the concentration in the adjacent bulk phase. Unlike absorption, which involves penetration of molecules into the bulk material, adsorption occurs within an interfacial layer formed between contacting phases, such as solid–liquid or solid–gas systems (Figure 2) [43,44,45]. This interfacial accumulation arises due to surface forces, including van der Waals forces and hydrophobic interactions, and is generally considered a reversible process [43,44,45]. Adsorption-based staining has gained popularity due to its low cost, technical simplicity, and ease of implementation. In many adsorption-based protocols, MNPs are mixed with a solvent-dye solution, incubated briefly, and then separated by filtration or washing [21,46]. Because minimal equipment is needed, it is easily reproducible and adaptable to multiple polymer types. Surface adsorption is commonly performed with Nile Red [20,21], but it has been adapted for other fluorophores such as rhodamine B [29], fluorescein [30], rhodamine 6G [30,32], and iDyes [40,41] as well.

An important consideration when using adsorption-based staining in biological systems is the potential for dissociation and off-target binding. For example, Nile Red may dissociate from polymer surfaces and bind to lipid-rich structures [47,48]. Differences in chemical composition, polarity, crystallinity, and surface properties influence dye-polymer interactions and staining efficiency [49]. For example, PE and PP are highly non-polar, whereas polyamides have greater polarity and hydrogen-bonding capacity [49]. These differences explain why dyes exhibit variable staining performance across different plastic types [31,50]. An additional limitation is that most staining protocols have been optimized for micro- rather than nanoplastics, resulting in a relative lack of validated methods for nanoscale particles. Nanoplastic staining differs due to size, surface effects, aggregation, optical resolution constraints, and nonspecific interactions, all of which can undermine adsorption-based fluorescence methods [51]. Currently, Nile Red remains the primary dye used for fluorescent labeling of MNPs, with most studies using PS as the model plastic. This narrow focus limits the range of validated staining strategies available for other environmentally relevant plastic types [28]. Overall, passive adsorption methods provide a rapid and technically simple initial approach for evaluating dye-polymer compatibility and determining whether sufficient fluorescence intensity can be achieved for microscopy-based analysis.

#### 2.3.2. Thermal Expansion and Swelling Diffusion

**Figure 3:**
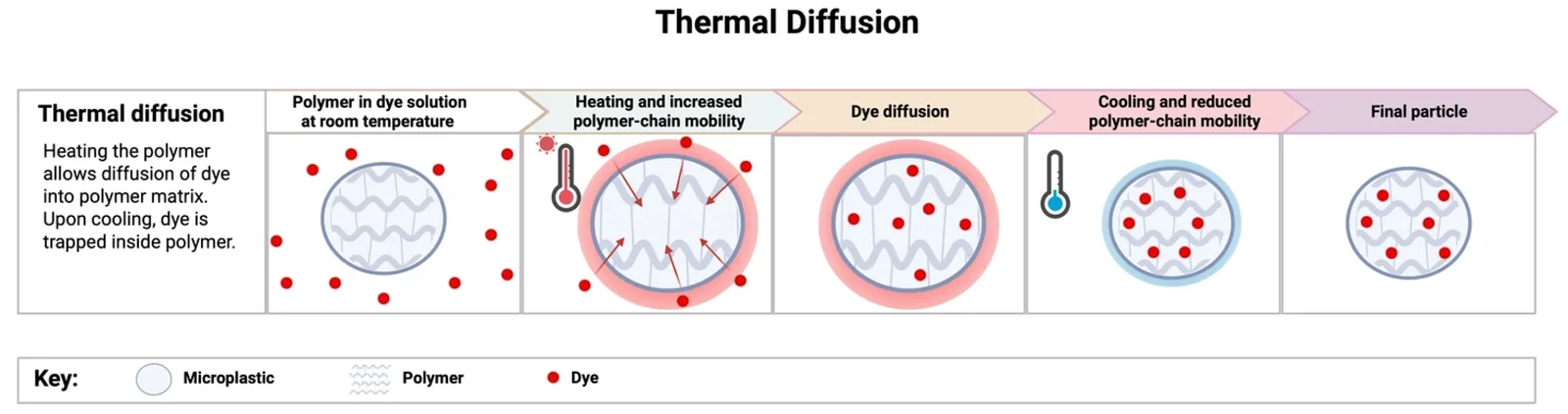
Heating promotes incorporation of dye within the polymer.

Thermal diffusion-based staining strategies involve heating plastic particles to increase polymer-chain mobility and free volume, enabling dye molecules to diffuse into the polymer matrix where they become trapped upon cooling (Figure 3) [52]. As polymers approach their glass transition temperature, the matrix changes from a rigid state to a rubber-like state that alters its capacity to accommodate and retain dye molecules [53]. This mechanism contrasts with conventional adsorption-based staining in which dye molecules are primarily confined to the polymer surface and are therefore more susceptible to desorption. Heat-assisted staining has been used with Nile Red, iDye, and Safranine T to fluorescently label less hydrophobic plastics at temperatures of approximately 60–70 °C [19,41,42,54].

**Figure 4:**
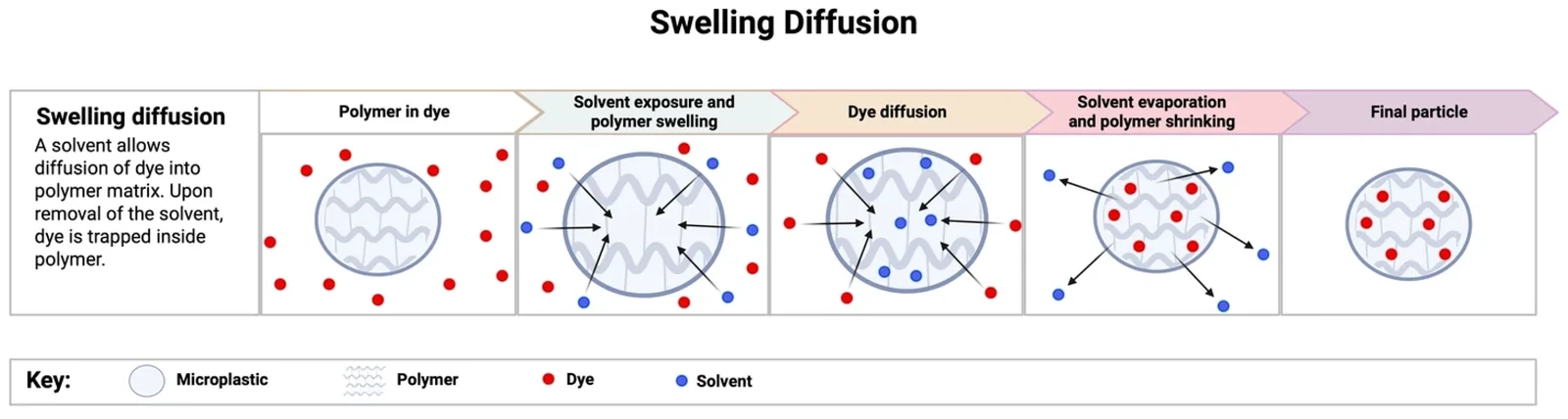
Polymer swelling enables dye to diffuse into the polymer matrix.

Alternatively, solvent-induced swelling diffusion relies on penetrant uptake, in which a compatible solvent enters the polymer matrix and induces swelling and plasticization (Figure 4) [55,56,57]. The expansion increases local polymer chain mobility and free volume, enabling dye molecules to diffuse into the polymer interior without thermal activation. For example, tetrahydrofuran can be used to swell PS MNPs and encapsulate hydrophobic fluorescent dyes [58]. This labeling approach has been applied to PS latex microspheres, which showed that the dye remained trapped within the polymer matrix after two years of storage in water [59].

The ability to inhibit leaching provides a direct advantage over adsorption-based techniques. The encapsulation of the dye molecules within the plastic further protects it from environmentally mediated degradation. Furthermore, since incorporation of the dye may limit surface modification, swelling-based dye deposition techniques produce fluorescent micro- and nanoplastics that are chemically and physically similar to their unlabeled polymer counterpart. To date, this technique has been shown to be highly compatible with many fluorescent dyes and polymer types [41,60,61]. By reducing surface-associated dye and minimizing leaching, swelling-based methods offer stable fluorescence properties.

#### 2.3.3. Covalent Fluorophore Conjugation

**Figure 5:**
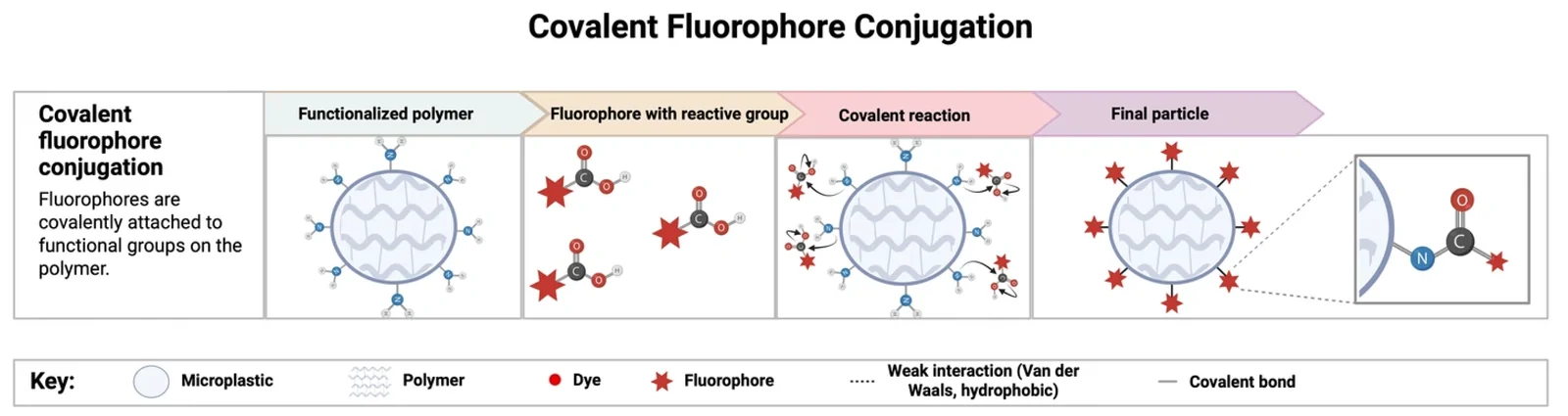
Stable fluorescent labeling is achieved through covalent attachment of fluorophores.

Because noncovalent interactions are susceptible to dye leaching in biological matrices, researchers frequently utilize covalent linkages to ensure much more stable fluorophore attachment (Figure 5). These covalent bonds provide substantially greater stability than noncovalent interactions, making them ideal for long-term tracking of polymeric materials [62,63]. Carboxyl-functionalized PMMA nanoparticles have been covalently labeled with fluorescent dyes to produce stable probes with reduced leaching that are suitable for reliable cellular uptake assessment and long-term imaging in biological studies [64]. Other examples include the conjugation of N-hydroxysuccinimide-terminated Atto 647N to amine-functionalized PS MNPs [24] and the coupling of IRDye 800 ester derivatives to amine-functionalized MNP spheres [32]. Because covalent labeling strategies rely on strong chemical bonds and typically require controlled reaction conditions, covalently bound fluorophores are less likely to dissociate from the polymer surface. This results in fluorescence stability during imaging and minimizes the likelihood of signal artifacts or false-positive detection resulting from fluorophore leaching.

A key limitation of covalent labeling strategies is that they often require chemical modification of the plastic polymers, commonly through oxidation, plasma functionalization, or copolymerization to introduce reactive functional groups. These modifications alter the native chemical surface composition, charge density, and surface energy of the particles. Consequently, introduced surface functional groups (e.g., carboxylates or amines) and bulky fluorophores can alter particle aggregation, protein corona formation, and cell-membrane interactions through Coulombic repulsion or steric interference.

In such cases, the resulting fluorescent plastics may not be fully representative of the original material and may therefore incompletely model the behavior of environmental plastics. In addition, some studies have reported that functionalized amine- and carboxyl-functionalized MNPs may exhibit greater toxicity compared to unmodified particles [65,66,67], further complicating their use as representative materials in toxicological studies.

#### 2.3.4. Polymerization-Based Labeling

**Figure 6:**
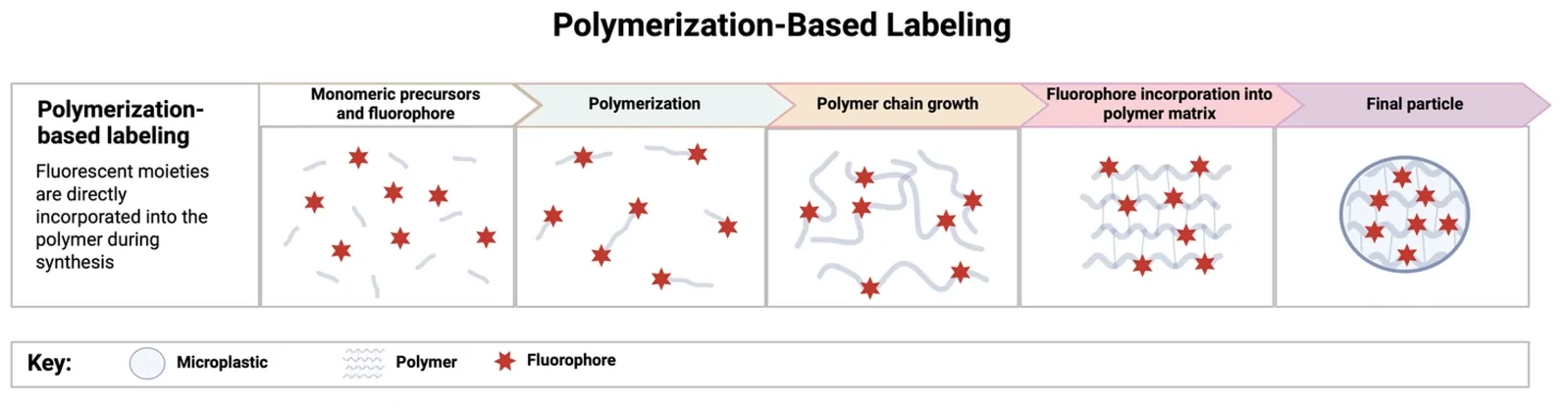
Fluorescent moieties are embedded within the polymer during synthesis.

Polymerization-based approaches incorporate fluorescent moieties directly into the polymer during synthesis, either through copolymerization with dye-functionalized monomers or by entrapment of fluorophores within the polymer matrix as it forms from monomeric precursors (Figure 6). In this approach, the fluorescent label becomes an integral part of the polymer structure rather than being introduced post-synthetically. This results in stable fluorescent particles with reduced leaching. This strategy can produce uniformly labeled MNPs with excellent photostability [28,68].

In one study, fluorescent PS MNPs were synthesized by dispersion polymerization of styrene with allyl-functionalized fluorescent dyes under nitrogen at 70 °C for 48 h. The resulting MNPs were isolated by precipitation, washed to remove any unbound dye, and dried under vacuum to yield stable fluorescent MNPs [69]. Similarly, fluorescent PMMA MNPs have been prepared by emulsion polymerization through copolymerization of methyl methacrylate with rhodamine-functionalized monomers in the presence of sodium dodecyl sulfate. Following synthesis, the surfactant was exchanged for the biocompatible surfactant Tween 80, yielding covalently fluorescent MNPs [70].

Despite these advantages, polymerization-based methods are limited by their technical complexity and relatively narrow applicability. In particular, these approaches often require specialized synthetic expertise and may not be readily compatible with all polymer types, including environmentally prevalent plastics such as PP and PE [28]. Ultimately, labeling strategies should be selected based on their intended application, please refer to Table 1 for a summary of the minimum validation needed to determine whether the fluorescent signal remains representative of the labeled particle.

**Table 1:** Qualitative classifications are comparative rather than universal. Performance depends on the fluorophore, polymer composition, particle size and morphology, weathering state, biological medium, exposure duration, and imaging platform. “Environmental relevance” refers to the extent to which the labeled particle retains the characteristics of environmentally encountered MNPs, not whether the method can detect particles in environmental samples.

Labeling strategy	Adsorption based staining [20,21,28,29,30,31,40,41,42,47,48,49,50,51]	Heat-assisted diffusion/thermal expansion [19,41,42,52,53,54]	Solvent-assisted swelling-diffusion [33,34,35,55,56,57,58,59,60,61]	Covalent fluorophore conjugation [24,32,62,63,64,65,66,67]	Fluorophore incorporation during polymerization [28,68,69,70]
Label stability	Low to moderate; dependent on dye-polymer affinity, washing, medium, and exposure duration	Moderate to high when the dye is retained within the polymer after cooling	Moderate to high; dyes incorporated into the polymer interior are generally retained better than surface-adsorbed dyes	High, provided the linkage remains chemically stable under the experimental conditions	Very high for covalently copolymerized fluorophores; high but formulation-dependent for physically entrapped dyes
Dye-leaching potential	High, particularly in lipid-rich or protein-containing biological media	Low to moderate; generally lower than passive adsorption but not necessarily eliminated	Low to moderate, depending on dye-polymer compatibility and the completeness of purification	Very low for intact covalent linkages; degradation or hydrolysis of the linker remains possible	Very low for covalently incorporated labels; low but potentially measurable for physically entrapped dyes
Polymer compatibility	Potentially broad, but strongly polymer- and dye-dependent; commonly applied to PE, PP, PS, PVC, PET, polyamide, and polyurethane	Limited to polymers that tolerate the required temperature; demonstrated for PE, PS, PVC, PET, and selected other polymers	Restricted by polymer-solvent-dye compatibility; demonstrated most extensively for PS and in selected PP, PE, and PET systems	Primarily applicable to polymers with native or introduced reactive groups, including amine- or carboxyl-functionalized PS and PMMA	Relatively limited; most established for synthetically accessible model polymers such as PS and PMMA
Suitability for in vitro studies	High for short-term screening and cellular-association studies when appropriate controls are included	High when particle morphology and dye retention are verified	High for cellular uptake, trafficking, and barrier-transport studies after removal of free dye and residual solvent	High for uptake, intracellular localization, and prolonged trafficking studies	High for controlled mechanistic studies requiring uniform and persistent fluorescence
Suitability for in vivo studies	Low for longitudinal tracking or biodistribution unless retention is demonstrated under the complete exposure conditions	Moderate; potentially suitable for short- or intermediate-duration studies following stability testing in biological media	Moderate to high when retention has been validated in relevant biological fluids and over the intended study duration	High, particularly with NIR fluorophores and validated linkage stability	High for longitudinal and biodistribution studies when the fluorophore is biocompatible and the particle is fully characterized
Preservation of particle physicochemical properties	Generally preserves bulk size and morphology, but adsorbed dye may alter surface hydrophobicity, charge, aggregation, or protein-corona formation	Moderate; heating may change crystallinity, morphology, surface properties, or additive release, particularly near glass-transition or melting temperatures	Moderate; swelling may alter particle size, morphology, porosity, aggregation, or surface chemistry, although optimized procedures can minimize these effects	Low to moderate; functionalization and conjugation can change surface charge, hydrophobicity, steric interactions, aggregation, protein-corona formation, and biological activity	Label retention is excellent, but the resulting particles are engineered materials whose size, shape, surface chemistry, and additive composition may differ substantially from environmental MNPs
Environmental relevance	Moderate to high, because environmentally derived particles can be labeled directly; interpretation may be compromised by dye desorption or selective staining	Moderate, because environmental particles can be used but may be altered by heating	Moderate, because irregular or environmentally derived particles may be labeled, but solvent treatment can alter weathered surfaces or extract additives	Low to moderate, because most environmental plastics lack the reactive surface groups required for conjugation	Low, particularly for uniform spherical particles synthesized from virgin monomers
Minimum recommended validation	Dye-only and unlabeled-particle controls; post-wash supernatant analysis; time-dependent leaching assessment in the exposure medium; pre- and post-labeling size and surface characterization	Temperature-matched unlabeled control; microscopy and size analysis before and after heating; leaching assessment; confirmation that heating does not substantially change particle morphology or aggregation	Free-dye and solvent-treated controls; exhaustive purification; residual solvent assessment; leaching studies in relevant media; pre- and post-labeling particle characterization	Purification and free-dye controls; linkage-stability testing; quantification of dye loading; comparison of size, morphology, zeta potential, and aggregation before and after conjugation	Confirmation of dye incorporation and absence of free dye; long-term stability testing; complete physicochemical characterization; comparison with environmentally relevant particles where extrapolation is intended

### 2.4. Modalities for Fluorescent MNP Detection

#### 2.4.1. Microscopy

Microscopy enables detection, localization, and semi-quantitative analysis of fluorescently labeled MNPs across biological systems [41,71,72]. Conventional wide-field fluorescence microscopy remains one of the most accessible approaches, supporting rapid visualization of labeled particles in environmental and biological samples. However, its limited axial resolution may constrain three-dimensional analysis [73]. Confocal microscopy addresses this limitation by improving optical sectioning and depth resolution, allowing for more precise analysis of particle distribution within tissues and cellular compartments [73,74]. Despite these advantages, both wide-field and confocal systems are limited by the diffraction barrier, such that nanoplastics typically appear as punctate signals, which restrict accurate size determination and detailed morphological characterization.

Advanced super-resolution techniques, such as stimulated emission depletion (STED) microscopy, can overcome this constraint by achieving sub-diffraction spatial resolution, enabling visualization of nanoplastics [24]. These approaches provide important insights into particle localization at smaller scales but require specialized instrumentation and are constrained by photobleaching, fluorophore compatibility, and trade-offs between resolution, signal intensity, and acquisition speed [75,76].

Multimodal imaging strategies further enhance analytical specificity. Correlative light and electron microscopy (CLEM), for example, integrates fluorescence-guided localization with ultrastructural imaging, enabling high-resolution confirmation of particle morphology [77]. When combined with complementary analytical techniques such as Raman spectroscopy, these workflows can improve confidence in particle identification while reducing the need for exhaustive imaging [78].

#### 2.4.2. Flow Cytometry

Flow cytometry provides a high-throughput approach for detecting and quantifying fluorescently labeled MNPs by measuring optical and fluorescence signals from individual particles in suspension. Although traditionally used for single-cell analysis, it can be adapted for fluorescent MNP detection [79,80]. In most studies, dyes such as Nile Red are used for rapid staining [81,82], enabling efficient detection and analysis of particles, often with short acquisition times. This approach is especially useful for particles larger than 200 nm and allows high-throughput, semi-quantitative assessment [81] which can be adapted to cell culture models to assess MNP uptake and internalization [83]. Imaging flow cytometry combines conventional flow cytometry with image-based confirmation of particles to support assessment of MNP uptake and cell-particle interactions in biological systems [84].

#### 2.4.3. In Vivo Imaging Systems and Near-Infrared Fluorescence Imaging

In vivo imaging systems (IVIS) utilize luminescence or fluorescence techniques to provide a noninvasive approach for visualizing and quantifying biological processes in living cells and animal models over time. This approach enables longitudinal assessment of disease progression, cell tracking, gene expression, micro- and nanoparticle biodistribution, and therapeutic response. Fluorescence-based analysis of MNPs in biological tissues may be limited by tissue autofluorescence and optical scattering, particularly at visible wavelengths between 400 and 550 nm. Near-infrared (NIR) fluorophores offer improved tissue penetration and reduced background signal for both in vivo and ex vivo detection. The advantages are readily applicable to MNP research [85,86,87], with in vivo studies demonstrating their strong potential for biological tracking, biodistribution assessment, and fluorescence-based particle analysis [32,88,89].

A variety of NIR fluorescence imaging devices are used in clinical studies; however, many of these platforms are not readily compatible with small-animal research [90]. Although primarily optimized for imaging within the NIR-I range, the IVIS Spectrum and IVIS Lumina platforms are among the most widely used small-animal imaging systems with NIR capability [91,92]. Additional platforms include the OV-110 Small Animal Imaging System [93], fluorescence molecular tomography [94], and photoacoustic imaging [95,96], the latter of which can be adapted for imaging within the NIR-II range.

For in vitro assays, NIR-I fluorescence can be detected using conventional fluorescence microscopes and plate readers equipped with appropriate excitation sources and emission filters. In contrast, NIR-II fluorescence detection typically requires specialized short-wave infrared instrumentation, often incorporating indium gallium arsenide detectors that may require liquid-nitrogen cooling. These detectors can be integrated into adapted confocal and light sheet microscopy systems to enable NIR-II fluorescence imaging [97].

#### 2.4.4. Raman Spectroscopy and FTIR

Vibrational spectroscopy, particularly Fourier-transform infrared (FTIR) and Raman spectroscopy, has been used to detect and chemically characterize MNPs in organic tissue samples [4,5,6]. FTIR spectroscopy measures the wavelength-dependent absorption of infrared radiation associated with molecular vibrations, producing a characteristic spectrum that can be compared with reference polymer libraries. Raman spectroscopy instead measures the inelastic scattering of monochromatic light, with the resulting frequency shifts reflecting the vibrational modes of the analyzed material [7,8]. Although the two techniques rely on different physical processes, both provide polymer-specific spectral fingerprints that can distinguish suspected plastic particles from biological material and other contaminants.

Each technique presents limitations when applied to organic tissues. The spatial resolution of conventional micro-FTIR analysis generally limits reliable identification of particles smaller than approximately 10–20 µm, although the practical limit depends on the instrument configuration, polymer, particle morphology, and surrounding matrix [9]. Water and residual biological material can produce strong IR absorption and overlapping spectral features that complicate FTIR analysis. Raman spectroscopy generally provides greater spatial resolution and can analyze smaller particles. However, fluorescence from tissue components, pigments, additives, or contaminants may overwhelm the comparatively weak Raman signal [9]. Sample processing is therefore commonly required to isolate suspected particles or reduce interfering biological material before spectroscopic analysis.

Fluorescence microscopy can complement vibrational spectroscopy by rapidly localizing suspected MNPs and directing subsequent analysis to selected regions of interest. Nile Red staining has been used as a screening strategy to identify candidate particles or particle-containing regions for targeted Raman analysis, reducing the number of locations requiring time-intensive spectroscopic interrogation [12,98,99]. NIR fluorophores may offer an additional advantage for biological imaging because excitation and emission at longer wavelengths can reduce interference from visible-spectrum tissue autofluorescence [100]. Nevertheless, neither Nile Red nor NIR fluorescence establishes polymer identity or confirms that the detected signal remains with an intact particle. Fluorescence-guided detection in organic tissues should therefore be supported by appropriate dye-only and unlabeled controls, assessment of dye retention, and confirmatory polymer identification using Raman, FTIR, or another chemically specific analytical method. Table 2 summarizes the various capabilities of different fluorescent imaging and analysis tools.

### 2.5. Nanoplastic-Specific Considerations for Fluorescent Labeling

Nanoplastics present experimental challenges that cannot be addressed simply by applying methods developed for larger microplastics. Their high surface-area-to-volume ratio, colloidal behavior, sub-diffraction dimensions, and strong interactions with biomolecules influence both behavior of the particles and the interpretation of particle-associated fluorescence [51,66,67]. Furthermore, nanoplastic research remains dominated by commercially manufactured PS spheres with uniform size and surface chemistry. Although these materials are experimentally convenient, their behavior may differ substantially from that of irregular, weathered, and chemically heterogeneous nanoplastics generated by environmental fragmentation.

#### 2.5.1. Colloidal Stability, Aggregation, and Protein Corona Formation

Nanoplastic dispersions are dynamic colloidal systems. Their aggregation state depends on particle size, surface charge, polymer composition, functionalization, pH, ionic strength, divalent-ion concentration, natural organic matter, and the composition of the exposure medium. Depending on these conditions, primary nanoplastics may form agglomerates whose hydrodynamic dimensions enter the micrometer range and whose increased settling alters the population available for exposure [101,102,103,104,105,106]. Adsorbed proteins or inorganic constituents may stabilize some suspensions through electrostatic or steric repulsion. However, charge screening, cation bridging, protein bridging, or inorganic particle bridging can instead promote aggregation under other conditions [101,102,103,104,105,106]. Consequently, the nominal size reported by a manufacturer or measured in water may not represent the size distribution or agglomeration state of the particles delivered to cells or tissues [107].

Aggregation and agglomeration can confound fluorescence-based particle counting and can alter cellular association and uptake, the effective dose delivered to cells, biodistribution, and toxicological responses [108,109,110]. A diffraction-limited punctum may represent a primary particle, an agglomerate containing multiple particles, or released fluorophore associated with cells, proteins, lipids, or tissue residues [24,26,27,110]. Nanoplastic studies should therefore characterize hydrodynamic diameter, polydispersity, zeta potential, and particle number or mass concentration in the actual exposure medium rather than exclusively in water or the original stock suspension [24,107,111].

When nanoplastics enter protein-containing biological media, proteins and other biomolecules adsorb to their surfaces and form a corona that establishes the particle’s biological identity [112,113,114]. Corona composition depends on particle size, polymer and surface chemistry, functionalization, morphology, and the composition and exposure history of the surrounding biological fluid. Protein adsorption can change the hydrodynamic size, zeta potential, aggregation behavior, membrane interactions, cellular uptake, epithelial transport, and biodistribution [108,109,112,113]. Biological matrices may also alter the fluorescent readout through fluorophore leaching, binding of released dye to biological material, or changes in the emission of environment-sensitive fluorophores [26,27,110,115].

#### 2.5.2. Detection Limits and Intracellular Localization

Nanoplastics below approximately 200–300 nm are smaller than the lateral diffraction-limited resolution of conventional fluorescence microscopy. They therefore appear as diffraction-limited spots corresponding to the microscope’s point-spread function rather than as spatially resolved particles [24,26,116]. Consequently, particle diameter and morphology cannot be determined reliably from the apparent dimensions of fluorescent spots alone. Fluorescence intensity is also not necessarily proportional to particle number because signal brightness depends on particle size and volume, fluorophore loading and distribution, fluorophore quantum yield, the local chemical environment, aggregation and agglomeration, self-quenching, acquisition settings, and photobleaching [36,110,116,117]. Aggregation can increase the fluorescence associated with an individual spot while decreasing the apparent number of discrete objects, whereas dye self-quenching or environmental quenching may reduce fluorescence [36,110,117].

Similarly, conventional flow cytometry has instrument-specific lower limits of detection and may not reliably distinguish small nanoplastics from electronic or optical noise, coincident or swarm events, dye aggregates, and particles present in reagents or exposure media [82,118,119,120]. Reliable measurements therefore require instrument-specific scatter and fluorescence calibration, filtered procedural blanks, unstained-particle and dye-only controls, threshold characterization, dilution series to test for coincident detection, and confirmation that detected events remain above the relevant noise floor [82,118,119,120].

Super-resolution fluorescence microscopy, fluorescence-lifetime imaging microscopy, and single-particle-tracking methods can improve nanoplastic detection, localization, and discrimination from biological autofluorescence [24,36,116,121]. For example, single-particle fluorescence microscopy has enabled the localization of fluorescent PS nanoplastics as small as approximately 50 nm in *Caenorhabditis elegans* [24], while single-particle tracking has been used to estimate the size and concentration of fluorescent plastic particles down to approximately 45 nm through analysis of Brownian motion [116]. STED microscopy has been shown to resolve labeled plastic particles smaller than 200 nm, and fluorescence-lifetime imaging can help distinguish labeled plastics from endogenous fluorescence based on fluorescence decay rather than intensity alone [36,121]. Nevertheless, these techniques detect fluorophore-associated signals and do not independently establish polymer identity or demonstrate that the fluorophore remains associated with an intact nanoplastic particle. Dye desorption, transfer to biological structures, and the formation of fluorescent dye aggregates can therefore produce false-positive particle detections unless dye-only, leachate, and unlabeled-particle controls are included.

Intracellular localization presents an additional challenge because particles adsorbed to the plasma membrane or positioned above or below a cell within the axial resolution of the microscope can be misclassified as internalized particles in two-dimensional images [112,122,123]. Confocal z-stacks, orthogonal image planes, membrane segmentation, and three-dimensional reconstruction improve spatial assignment but do not overcome the optical-resolution limit when particle dimensions and particle-to-membrane distances are smaller than the effective resolution of the imaging system [112,122,123]. Evidence for internalization can be strengthened through extracellular-fluorescence quenching, rigorous removal of surface-bound particles, orthogonal image planes, three-dimensional segmentation, and co-localization with endosomal or lysosomal markers [122,123]. CLEM or complementary non-fluorescent particle tracers can provide additional confirmation of intracellular particle localization [124,125].

### 2.6. Validation and Reporting Framework

Fluorescence provides a sensitive means of detecting and localizing MNPs, but it directly measures the fluorophore rather than the polymer itself. Consequently, fluorescence associated with a biological sample cannot, by itself, establish the presence, identity, number, or location of intact MNPs. Released dye, dye aggregates, biological autofluorescence, nonspecific binding, particle aggregation, and matrix-dependent changes in fluorescence can all produce signals that are incorrectly attributed to particles [26,27,28]. These concerns apply to both microplastics and nanoplastics but become especially important at the nanoscale, where individual particles cannot generally be resolved by conventional fluorescence microscopy. A rigorous validation workflow should connect the intended biological inference to the labeling method, particle characterization, control conditions, analytical performance, and degree of orthogonal confirmation required.

#### 2.6.1. Define Inference and Labeling Strategy

Validation requirements should first be determined by the experimental question. Fluorescence used to screen environmental samples for suspected microplastics requires different evidence than fluorescence used to demonstrate cellular internalization, barrier crossing, or whole-animal biodistribution. Adsorption-based staining may be sufficient for rapid screening or short-term localization when appropriate controls and confirmatory polymer identification are included. In contrast, prolonged cellular trafficking, barrier transport, and in vivo biodistribution studies generally require more stable labels produced through swelling diffusion, covalent conjugation, or incorporation during polymerization [19,20,21,22,23,24,28,126,127,128,129].

The labeling method should be validated separately for the polymer, particle dimensions, morphology, weathering state, exposure medium, and study duration being investigated. Dye performance demonstrated with pristine PS spheres, for example, should not be assumed to apply to irregular, weathered PE or PP particles. Similarly, evidence of fluorescence retention in water does not demonstrate stability in serum-containing culture medium, simulated gastrointestinal fluids, plasma, or tissue. The rigor of validation should increase with the strength of the intended conclusion, particularly when fluorescence is used to support claims of intact particle internalization, translocation, or organ accumulation.

#### 2.6.2. Fluorophore Retention and Removal of Unbound Dye

The labeled particles should be purified using an appropriate particle-dye separation method until unbound fluorophore is no longer detectable or has been reduced to a predefined background level. Analysis of the final wash, post-purification supernatant, or particle-free filtrate can help determine whether residual free dye remains. However, successful removal of initially unbound fluorophore does not demonstrate that the retained label will remain associated with the particles throughout the experiment.

Label stability should therefore be evaluated over the complete experimental duration in the actual exposure medium. Relevant conditions include the anticipated pH, ionic composition, protein and lipid content, temperature, and exposure time because these factors can alter fluorophore desorption, linker stability, and transfer of dye to biological structures [26,27,28,126,127,130]. Measurements should distinguish particle-associated fluorescence from fluorescence present in the particle-free fraction at multiple time points. For noncovalently labeled particles, this assessment is particularly important because even a small released fraction may generate a prominent biological signal if the free fluorophore preferentially accumulates in membranes, lipid-rich compartments, cells, or tissues.

#### 2.6.3. Characterize Particles Before and After Labeling

Fluorescent labeling and subsequent purification may change the properties that govern MNP behavior. Particle size distribution, morphology, concentration, surface charge, aggregation state, and fluorescence characteristics should be evaluated before and after labeling. Dye loading and changes in surface hydrophobicity should also be assessed. These comparisons are particularly important for covalently labeled particles because the functionalization required for conjugation may alter surface charge, protein adsorption, cellular uptake, and biological activity [126,128,129,131].

For nanoplastics, characterization should be performed in the intended exposure medium rather than only in water or the original stock suspension. At minimum, studies should consider hydrodynamic diameter, polydispersity, zeta potential, aggregation or agglomeration, and particle number or mass concentration at baseline and at biologically relevant time points [24,107,110,111,132,133]. Measurements should be performed using complementary methods such as dynamic light scattering, nanoparticle-tracking analysis, asymmetrical-flow field-flow fractionation, and electron microscopy [110,111,132]. Preparation and handling conditions, including sonication energy and duration, dispersants, particle concentration, storage duration, and mixing procedures, should also be reported, as they can alter dispersion characteristics and experimental reproducibility [133].

For larger microplastics, microscopy can provide direct information about size and morphology, but labeling may still alter surface properties or selectively stain polymer types, particle sizes, or weathering states. Validation should assess whether the labeled and recovered particles remain representative of the original material and whether the labeling procedure introduces a systematic detection bias.

#### 2.6.4. Incorporate Controls and Establish Analytical Performance

A minimum control set should include unexposed biological controls, unlabeled-particle controls, fluorophore-only controls, exposure-medium and procedural blanks, and leachate or final post-purification supernatant controls [26,27,28,126,127,128]. Fluorophore-only controls should undergo the same incubation, purification, and imaging conditions as the labeled particle samples whenever feasible. Together, these controls help distinguish particle-associated fluorescence from biological autofluorescence, nonspecific dye binding, dye aggregates, contaminated reagents, and residual free fluorophore.

Analytical performance should be established separately for each relevant biological matrix. Reported parameters should include procedural-blank contributions, whole-method spike recovery, working range, signal linearity, repeatability or precision, and matrix-specific limits of detection and quantification [134,135]. Calibration materials should resemble the labeled particles and matrix used in the experiment because fluorescence intensity can be affected by particle size, aggregation, dye loading, tissue attenuation, quenching, photobleaching, and detector saturation [136].

Additional validation should be tailored to the detection platform. Flow-cytometry studies should characterize instrument-specific scatter and fluorescence thresholds, use filtered blanks, and perform dilution series to identify coincident or swarm detection [82,118,119,120]. Microscopy studies should document acquisition settings, spectral filters, background-subtraction procedures, and criteria used to define a particle-associated event. Whole-animal imaging and ex vivo optical imaging should account for tissue attenuation, organ-specific autofluorescence, and the possibility that free dye and labeled particles have different pharmacokinetic distributions.

#### 2.6.5. Orthogonal Confirmation of Particle Identity and Localization

The need for orthogonal confirmation depends on the intended conclusion. Fluorescence may be sufficient for comparative screening when label retention and analytical performance have been established. When polymer identity is central, suspected particles should be confirmed using a chemically specific method such as Raman or FTIR spectrometry [12,25,98,99]. Fluorescence-guided spectroscopy can improve efficiency by directing analysis toward candidate particles or regions of interest, but fluorescence should not substitute for polymer-specific identification.

For barrier transport and biodistribution studies, fluorescence should be combined with an orthogonal method whenever intact-particle translocation is a central conclusion. Raman-based methods can provide polymer-specific vibrational signatures under suitable analytical conditions, whereas pyrolysis–gas chromatography–mass spectrometry (Py-GC-MS) can identify and quantify polymer mass but does not preserve spatial or particle number information [134,137,138,139]. The selected confirmatory method should therefore match the particle dimensions, biological matrix, and specific claim being evaluated.

Studies should report the labeling procedure, purification method, dye-loading or fluorescence characteristics, evidence of label retention, pre- and post-labeling particle properties, control conditions, calibration approach, imaging parameters, recovery, and detection limits. Applying this structured workflow will improve comparability among studies and reduce the risk that fluorophore behavior, rather than MNP behavior, is interpreted as evidence of particle uptake, transport, or accumulation.

### 2.7. Biological Applications of Fluorescent MNPs

Fluorescent labeling is particularly valuable in biological MNP research because it enables spatially and temporally resolved tracking of particles within complex cellular and tissue environments. However, the strength of the biological inference depends on whether the fluorescent signal remains associated with the MNP throughout the experiment. Laboratory-produced MNPs often lack the weathering, UV exposure, microbial interactions, and other surface transformations experienced by plastics in the environment. The labeling strategy should therefore be selected according to the intended biological application while accounting for how differences between laboratory and environmentally derived particles may affect experimental relevance. Short-term in vitro screening may be compatible with adsorption-based approaches when adequate washing and dye-only controls are used, whereas studies of prolonged cellular trafficking, barrier transport, or whole-animal biodistribution generally require more stable labeling approaches.

#### 2.7.1. Cellular Uptake and Intracellular Localization

Fluorescently labeled MNPs enable direct visualization of particle association with cells, intracellular localization, and redistribution over time in cell-culture models. Fluorescence microscopy and confocal microscopy can be used to help differentiate particle signal associated with the cell surface from signal located within the cellular volume, while co-labeling with nuclear, lysosomal, endosomal, or membrane markers can provide information regarding intracellular trafficking [140]. Fluorescent MNPs of different sizes, polymer types, or surface chemistries can also be compared within the same experimental system to assess material- and size-dependent differences in cell association and uptake [141].

For example, fluorescent PE microplastics measuring 1–4 µm were transported across an intestinal epithelial cell monolayer to a small but significant extent and at greater levels than similarly sized PS particles [142]. In rat basophilic leukemia cells, fluorescent 50 nm and 500 nm PS particles were internalized through size-dependent pathways, accumulated predominantly in lysosomes, and were subsequently released through passive transport and lysosomal exocytosis, whereas 5 µm particles showed little internalization [140]. In THP-1-derived macrophages, flow cytometry and confocal microscopy further demonstrated that a gastrointestinal digestion-associated protein corona increased uptake of uncharged PS particles smaller than 500 nm by approximately four- to six-fold, illustrating how acquired surface layers can modify cellular interactions [113].

#### 2.7.2. Barrier Transport and In Vivo Biodistribution

Fluorescent and other optical labeling approaches can support investigation of MNP transport across biological barriers and subsequent tissue distribution in animal models. In contrast to endpoint chemical analysis, optical imaging can provide spatially resolved and, in some cases, longitudinal information regarding particle-associated signals in living animals, excised tissues, or histologic sections. These capabilities have been used to investigate MNP passage across intestinal, pulmonary, placental, and blood-brain interfaces, as well as subsequent distribution to secondary organs [143,144,145,146].

Fluorescence-based biodistribution studies have demonstrated the utility of this approach across multiple model systems. In mice orally exposed to PP MNPs labeled with Cy5.5–COOH, most of the administered material was reported to be excreted through the gastrointestinal tract within 24 h [33]. Similar fluorescence-enabled tracking has been applied in zebrafish embryos exposed to rhodamine B-labeled MNPs [60]. In another study, rhodamine-labeled PS nanoplastics administered by inhalation were detected beyond the pulmonary compartment and within fetal tissues, including the liver, lungs, and brain [147]. Together, these studies illustrate the value of fluorescent labeling for mapping particle-associated signal across tissues and time points.

For in vivo applications, stable labeling is especially important because fluorescent signal detected in tissues may not always represent intact MNPs. Dye leaching, tissue autofluorescence, and nonspecific fluorophore binding can generate apparent translocation or tissue accumulation [26]. Covalently bound, matrix-embedded, or polymerization-incorporated fluorophores, as well as NIR labels for deep-tissue imaging, may therefore be preferable for studies requiring prolonged circulation, biodistribution analysis, or whole-animal imaging. Orthogonal confirmation by Raman spectroscopy, electron microscopy, or chemical analysis can further strengthen interpretation when tissue localization is a primary endpoint.

#### 2.7.3. Relative Quantification and Longitudinal Burden Assessment

Fluorescence can also support semi-quantitative assessment of relative MNP burden across biological samples. Flow cytometry integrates fluorescence and light-scattering measurements to identify and compare labeled particles or particle-associated cellular events in suspension [148]. In cell culture, this approach can be used to compare relative uptake between experimental conditions, whereas imaging flow cytometry can provide visual confirmation of cell-particle association [149,150]. Similarly, fluorescence microscopy and whole-animal optical imaging can be paired with standard curves generated from known concentrations of fluorescent MNPs to estimate relative tissue-associated signal [151].

These approaches should be interpreted as comparative rather than absolute measures unless particle-specific calibration and recovery experiments have been performed. Fluorescence intensity can be influenced by particle size, particle aggregation, dye loading, tissue attenuation, photobleaching, and signal saturation at higher concentrations [136]. Consequently, fluorescence-derived estimates of MNP accumulation should be reported alongside the labeling method, excitation and emission settings, calibration strategy, relevant biological controls, and evidence for dye retention under the experimental conditions used. This framework will improve the reliability of fluorescence-based comparisons and clarify the extent to which measured signal reflects intact labeled MNPs.

#### 2.7.4. Consideration of Environmentally and Biologically Acquired Surface Transformations

The surfaces of MNPs are dynamic and can change substantially between environmental release, biological exposure, and analytical selection. UV photooxidation, mechanical abrasion, additive leaching, adsorption of natural organic matter and contaminants, and microbial colonization can alter particle roughness, oxidation, hydrophobicity, charge, aggregation, and transport behavior [152,153,154,155,156]. Dissolved organic matter may rapidly form a conditioning layer, or eco-corona, that modifies the polymer-water interface and influences subsequent biofilm development [157]. These transformations can change particle wettability, density, contaminant adsorption, and interactions with biological molecules. Consequently, pristine laboratory particles may behave differently from environmentally conditioned particles even when they have the same polymer composition and nominal dimensions.

Upon contact with biological fluids, proteins, lipids, metabolites, and other biomolecules adsorb to MNP surfaces to form a biomolecular corona that establishes the identity presented to cells. Corona composition depends on polymer type, particle size and morphology, surface charge, functionalization, environmental history, and the surrounding biological medium [108,109,112,113,114]. Ducoli et al. found that environmentally representative nanoplastics acquired a different protein corona than commercial PS nanobeads [114], while Dawson et al. demonstrated that polymer composition, morphology, additives, and aqueous leaching influenced protein adsorption to microplastic beads, fibers, and fragments [155]. These changes can affect colloidal stability, membrane interactions, cellular uptake, intracellular trafficking, clearance, and biodistribution. For example, a gastrointestinal digestion-associated corona persisted after transfer of PS MNPs into serum-containing medium and increased macrophage uptake of uncharged particles smaller than 500 nm by approximately four- to six-fold [113]. Other studies have shown that plasma-corona formation can alter nanoplastic intracellular trafficking and biochemical effects [158] and that biomolecular-corona composition may influence interactions with the blood–brain barrier [146].

Surface transformations may also affect fluorescence performance. Environmental coatings and biological coronas can change particle aggregation, local polarity, light scattering, and the chemical environment surrounding a fluorophore, potentially altering its fluorescence intensity or emission characteristics. Biofilms and adsorbed biomolecules may introduce additional autofluorescence, mask surface-associated dyes, or facilitate dye transfer to proteins and lipids. Conversely, fluorescent labeling may itself change the surface properties governing corona formation. Commercially fluorescently labeled PS particles have been shown to differ from unlabeled particles in hydrophobicity, plasma-protein adsorption, and phagocytic uptake [131]. Protein-corona formation has also been reported to reduce the fluorescence intensity of labeled amine-functionalized PS nanoplastics, demonstrating that changes in measured signal do not necessarily indicate proportional changes in particle abundance [158].

### 2.8. Remaining Challenges and Research Priorities in Fluorescent MNP Research

The validation workflow described in Section 2.5 can reduce misinterpretation of fluorescence-based findings, but several fundamental challenges remain. These include the tradeoff between label stability and particle representativeness, optical and biological confounding, limited environmental relevance of model particles, and the absence of standardized methods across studies. Addressing these challenges will require not only improved validation within individual experiments but also broader methodological harmonization across the field.

A central challenge is that strategies used to improve fluorophore retention may alter the properties that determine MNP behavior. Adsorption-based staining can be applied directly to environmentally derived particles and may preserve their bulk size and morphology, but the label remains susceptible to desorption and transfer to biological structures. This can confound fluorescence-based measurements and contribute to false-positive detection of MNPs [26,130,159,160]. Conversely, thermal- or solvent-assisted incorporation, covalent conjugation, and polymerization-based labeling can improve fluorescence stability but may alter particle crystallinity, porosity, surface charge, hydrophobicity, aggregation, or protein-corona formation [28,65,66,67,68,69,70,126,129,131].

Fundamental optical limitations also constrain fluorescent MNP analysis. Photobleaching causes progressive and irreversible signal loss during prolonged or repeated imaging, potentially affecting longitudinal comparisons [117,161,162]. Fluorescence intensity is also influenced by dye loading, local chemical environment, self-quenching, particle aggregation, acquisition settings, tissue attenuation, and biological autofluorescence [26,36,116,136]. These factors prevent fluorescence intensity from being interpreted directly as particle number or mass without particle- and matrix-specific calibration. The problem is particularly pronounced for nanoplastics, which generally appear as diffraction-limited puncta rather than spatially resolved particles under conventional fluorescence microscopy. Even with appropriate validation, fluorescence alone cannot determine whether an individual punctum represents a primary nanoplastic, an agglomerate, released fluorophore, or fluorescent material associated with a nearby biological structure.

Fluorescent labels and the chemical modifications required for labeling may also influence biological responses. Leached dyes can exert toxic effects independently of the plastic particle, while particle functionalization can alter cellular uptake, membrane interactions, protein adsorption, and toxicity [65,66,67,130,131,163,164]. These effects are especially important in toxicological studies because an observed response may reflect the plastic, the fluorophore, residual labeling reagents, labeling-induced changes in particle properties, or a combination of these factors. The controls described in Section 2.6.4 can help distinguish these contributions, but they cannot fully restore the behavior of an unlabeled particle if the labeling procedure has changed its biological identity.

Environmental representativeness remains another major limitation. Much of the existing biological literature relies on commercially produced fluorescent PS spheres that are uniform in size, shape, and surface chemistry and may contain weakly bound dyes that can leach into biological systems [26]. Environmental MNPs are often heterogenous in size, morphology, polymer composition, and surface chemistry. They may also undergo ultraviolet-induced weathering [152,153], accumulate microbial biofilms and adsorbed proteins [154,155,156], and contain diverse additives with known or suspected toxic potential [165,166]. These processes can influence particle transport, aggregation, cellular uptake, biodistribution, and toxicity. Commercial fluorescent particles are therefore useful for controlled mechanistic studies but may incompletely model real-world exposure. Conversely, labeling environmentally derived particles can introduce selective detection because fluorophore retention varies across polymers, particle sizes, morphologies, and weathering states. A fluorescently detected particle population may consequently represent only the fraction of MNPs that was successfully labeled and recovered.

Finally, comparison among studies remains difficult because labeling protocols, particle-characterization methods, exposure media, stability assessments, imaging settings, and reporting practices vary substantially. The absence of broadly accepted fluorescent MNP reference materials and harmonized performance criteria limits reproducibility and prevents direct comparison of detection limits, uptake measurements, and biodistribution estimates across laboratories [28,128,129,134,135]. Future research should prioritize interlaboratory validation, polymer- and matrix-specific reference materials, standardized reporting of labeling and imaging parameters, and consensus criteria for demonstrating label retention and intact particle detection. Until such standards are established, fluorescence is most defensible as a sensitive localization and comparative tool incorporated into a multimodal analytical workflow rather than as independent proof of MNP identity, quantity, internalization, or translocation.

## 3. Conclusions

Fluorescent labeling of MNPs can be helpful for in vivo and in vitro laboratory studies, enabling detection at the cellular, organ, and whole-body level. Fluorescence is most useful as a sensitive localization and comparative tool for MNPs, but it should not be treated as independent proof of particle identity, abundance, internalization, or translocation. The strongest labeling strategy depends on the intended inference. Nile Red-based adsorption remains the most established and practical approach for rapid screening of microplastics, but its susceptibility to nonspecific staining and dye transfer limits its use in biological systems and requires polymer-specific confirmation. For prolonged cellular-trafficking and biodistribution studies, matrix-embedded, covalent, and polymerization-based labels provide substantially greater signal retention than surface adsorption. Swelling-diffusion methods may offer the best practical compromise between label stability and preservation of particle-surface properties, whereas covalent conjugation and incorporation during polymerization provide the strongest attachment but produce more highly engineered particles. Near-infrared labels are particularly advantageous for whole-animal and ex vivo imaging because they reduce tissue autofluorescence and improve signal detection at depth. Nevertheless, no labeling method has demonstrated universal reliability across polymer types, particle dimensions, biological matrices, and exposure durations.

The principal unresolved challenge is the tradeoff between fluorescence stability and particle representativeness. Weakly associated dyes can leach, redistribute to lipid-rich biological structures, or generate false-positive signals, whereas the functionalization, heating, solvent exposure, or synthetic incorporation used to improve retention may alter particle size, surface charge, aggregation, protein-corona formation, cellular uptake, and toxicity. These concerns are compounded at the nanoscale, where conventional fluorescence microscopy cannot resolve individual particles and fluorescence intensity cannot be converted directly to particle number or mass without particle- and matrix-specific calibration. The continued reliance on uniform, commercially produced fluorescent polystyrene spheres further limits extrapolation to environmentally relevant MNPs, which are heterogenous, weathered, irregularly shaped, and dynamically modified by environmental and biological coronas. Consequently, studies using fluorescent model particles should distinguish controlled mechanistic findings from conclusions about real-world exposure.

Future research should therefore prioritize validation standards rather than the continued proliferation of incompletely characterized fluorescent probes. Labeling methods should be evaluated for fluorophore retention over the full experimental duration in the actual exposure medium; labeled particles should be characterized before and after labeling for changes in size, morphology, surface charge, aggregation, and concentration, and experiments should include unlabeled-particle, fluorophore-only, procedural-blank, and leachate controls. Matrix-specific recovery, detection limits, signal linearity, and imaging parameters should also be reported. Finally, claims of polymer identity, cellular internalization, barrier crossing, or organ accumulation should be supported by an orthogonal analytical method whenever feasible. Development of polymer- and matrix-specific reference materials, environmentally representative labeled particles, harmonized reporting criteria, and interlaboratory validation will be essential for transforming fluorescence-based MNP tracking from a largely qualitative approach into a reproducible and quantitatively defensible methodology.

## Figures and Tables

**Table 2:** Comparative capabilities of analytical and imaging modalities used with fluorescently labeled MNPs. FTIR, Fourier-transform infrared spectroscopy; NIR, near-infrared.

Modality	Spatial Resolution/Practical Detection Limit	Imaging Depth	Throughput	Major Advantage	Principle Limitation
Widefield fluorescence microscopy	• Approximately 200–300 nm lateral resolution • Smaller particles appear as unresolved puncta	• Thin specimens or near-surface signal	• Moderate to high	• Rapid, accessible localization over a large field of view	• Out of focus fluorescence • Limited axial localization
Confocal fluorescence microscopy	• Approximately 180–250 nm lateral and approximately 500–700 nm axial resolution	• Tens to hundreds of microns, depending on specimen and optics	• Low to moderate	• Optical sectioning, z-stacks, and three-dimensional localization	• Slower acquisition • Photobleaching • Inability to resolve most nanoplastics individually
Flow cytometry	• Instrument- and fluorescence-dependent • Optimized systems may detect particles below 200 nm, but no universal size cutoff applies	• N/A	• Very high	• Rapid quantitative analysis of large event populations	• Small particles may be confused with noise, dye aggregates, or coincident event • Limited spatial information
In vivo imaging systems (IVIS)	• Approximately submillimeter near the surface, degrading to several millimeters with depth • No universal particle-level detection	• Visible: superficial • NIR: several millimeters to approximately centimeters, depending on instrument and tissue	• Moderate to high	• Noninvasive longitudinal imaging • Rapid ex vivo organ screening	• Depth-dependent attenuation • Poor anatomical localization • Inability to distinguish intact particles from released dye
Micro-FTIR *	• Approximately 10–20 µm, although instrument-dependent	• Surface or thin-section analysis	• Moderate; higher with focal-plane arrays	• Polymer-specific chemical identification	• Limited sensitivity for small particles • Interference from water and biological matrix
Micro-Raman *	• Approximately 1 µm under favorable conditions	• Surface or thin-section analysis	• Low	• Polymer-specific identification at smaller sizes than micro-FTIR	• Slow acquisition • Weak Raman signal • Fluorescence interference

* Does not primarily rely on fluorescence for detection.

## Data Availability

No new data were created or analyzed in this study. Data sharing is not applicable to this article.
